# Intranasal administration of octavalent next-generation influenza vaccine elicits protective immune responses against seasonal and pre-pandemic viruses

**DOI:** 10.1128/jvi.00354-24

**Published:** 2024-08-22

**Authors:** Naoko Uno, Thomas Ebensen, Carlos A. Guzman, Ted M. Ross

**Affiliations:** 1 Center for Vaccines and Immunology, University of Georgia, Athens, Georgia, USA; 2 Department of Infection Biology, Lehner Research Institute, Cleveland Clinic, Cleveland, Ohio, USA; 3 Department of Vaccinology and Applied Microbiology, Helmholtz Centre for Infection Research, Braunschweig, Germany; 4 Florida Research and Innovation Center, Cleveland Clinic, Port Saint Lucie, Florida, USA; 5 Department of Infectious Diseases, University of Georgia, Athens, Georgia, USA; Emory University School of Medicine, Atlanta, Georgia, USA

**Keywords:** influenza, vaccine, COBRA, intranasal, Sting agonist, mice, ferret, multivalent

## Abstract

Development of next-generation influenza virus vaccines is crucial to improve protection against circulating and emerging viruses. Current vaccine formulations have to be updated annually due to mutations in seasonal strains and do not offer protection against strains with pandemic potential. Computationally optimized broadly reactive antigen (COBRA) methodology has been utilized by our group to generate broadly reactive immunogens for individual influenza subtypes, which elicit protective immune responses against a broad range of strains over numerous seasons. Octavalent mixtures of COBRA hemagglutinin (HA) (H1, H2, H3, H5, H7, and influenza B virus) plus neuraminidase (NA) (N1 and N2) recombinant proteins mixed with c-di-AMP adjuvant were administered intranasally to naive or pre-immune ferrets in prime-boost fashion. Four weeks after final vaccination, collected sera were analyzed for breadth of antibody response, and the animals were challenged with seasonal or pre-pandemic strains. The octavalent COBRA vaccine elicited antibodies that recognized a broad panel of strains representing different subtypes, and these vaccinated animals were protected against influenza virus challenges. Overall, this study demonstrated that the mixture of eight COBRA HA/NA proteins mixed with an intranasal adjuvant is a promising candidate for a universal influenza vaccine.

**IMPORTANCE:**

Influenza is a respiratory virus which infects around a billion people globally every year, with millions experiencing severe illness. Commercial vaccine efficacy varies year to year and can be low due to mismatch of circulating virus strains. Thus, the formulation of current vaccines has to be adapted accordingly every year. The development of a broadly reactive influenza vaccine would lessen the global economic and public health burden caused by the different types of influenza viruses. The significance of our research is producing a promising universal vaccine candidate which provides protection against a wider range of virus strains over a wider range of time.

## INTRODUCTION

Influenza viruses cause severe respiratory disease in millions of people, resulting in severe morbidity and mortality each year (1, 2). There are three types of influenza viruses that infect humans: A, B, and C (IAV, IBV, and ICV, respectively) (3). IAV is divided into two phylogenetic groups, group 1 and group 2 (4), and further distinguished into subtypes identified by the hemagglutinin (HA) and neuraminidase (NA) surface glycoproteins. Currently, there are 18 identified HA proteins (H1–H18) and 11 identified NA proteins (N1–N11) (2). IBV is classified into two lineages: Yamagata-like and Victoria-like (5). H1N1 and H3N2 IAV and IBV strains are circulating in humans, whereas avian strains of the H2, H5, and H7 subtypes have pandemic potential if transmitted from animal reservoirs to humans (6).

The effectiveness of current influenza virus vaccines varies from year to year, ranging from 10% to 60% (7). The vaccines are composed of three to four strains representing seasonal subtypes H1N1, H3N2, and IBV, which need to be updated annually (8, 9). Accurate predictions of circulating strains are difficult due to antigenic variations of the virus from constant mutations and immune selective pressures (10). An ideal universal influenza vaccine would provide protection against both circulating human strains and zoonotic strains with pandemic potential, lasting multiple seasons (11).

Recombinant proteins are a promising alternative to the split-inactivated or attenuated influenza virus vaccines that are the industry standard. Recombinant protein vaccines have similar vaccine effectiveness in healthy adults and increased effectiveness in the elderly or immunocompromised (12). Recombinant protein vaccines provide better protection against influenza-related hospitalization compared to split-inactivated vaccines in women and younger adults in general and in people without any high-risk conditions (13). Repeated recombinant protein vaccinations redirect antibody responses to effective epitopes and away from egg-adapted mutations since most current vaccines are produced in embryonated chicken eggs, which reduces the antigenicity and effectiveness of the vaccine (14).

Computationally optimized broadly reactive antigen (COBRA) methodology aligns multiple consensus sequences from thousands of isolates to generate a final immunogen that stimulates cross-protective immune responses against a large panel of strains for each subtype. Monovalent COBRA immunogens elicit wider breadth of immune response than wild-type (WT) comparators (15–20). Mice vaccinated with heptavalent vaccine formulations composed of COBRA HA and NA proteins formulated with the adjuvant, Addavax, elicited protective immune responses against both seasonal and pre-pandemic IAV strains when administered by intramuscular route (21).

Most licensed influenza vaccines are delivered intramuscularly, which elicit systemic immune responses, but not local mucosal immune responses (22). Intranasal vaccine delivery can elicit robust humoral and cellular responses not only at systemic levels but also at the site of influenza virus infection (23, 24). This can, in turn, be instrumental to reduce virus infection, reducing horizontal transmission to susceptible hosts. Intranasal delivery has practical advantages over intramuscular delivery with increased patient compliance, ease of administration, and reduced sanitary costs (25). Vaccines delivered to mucosal surfaces should be formulated with adjuvants in order to overcome tolerogenic immune response and degradation (26).

In this study, in order to expand the coverage of elicited immune responses to pre-pandemic and IBV strains, an octavalent vaccine composed of H1, H2, H3, H5, H7, and IBV COBRA HA proteins and N1 and N2 COBRA NA proteins was developed and tested in both immunologically naive ferrets and ferrets pre-immune to historical influenza viruses. The vaccine was administered intranasally following mixture with the stimulator of interferon gene (STING) activator, bis-(3,5)-cyclic dimeric adenosine monophosphate (c-di-AMP) that is a strong stimulator of mucosal immune responses (27). This octavalent COBRA HA/NA-based vaccine elicited protective immune responses against seasonal and pre-pandemic influenza viruses.

## RESULTS

### Vaccination with the octavalent COBRA-based vaccine elicits broadly reactive antibody activity against multiple influenza virus strains

**Fig 1:**
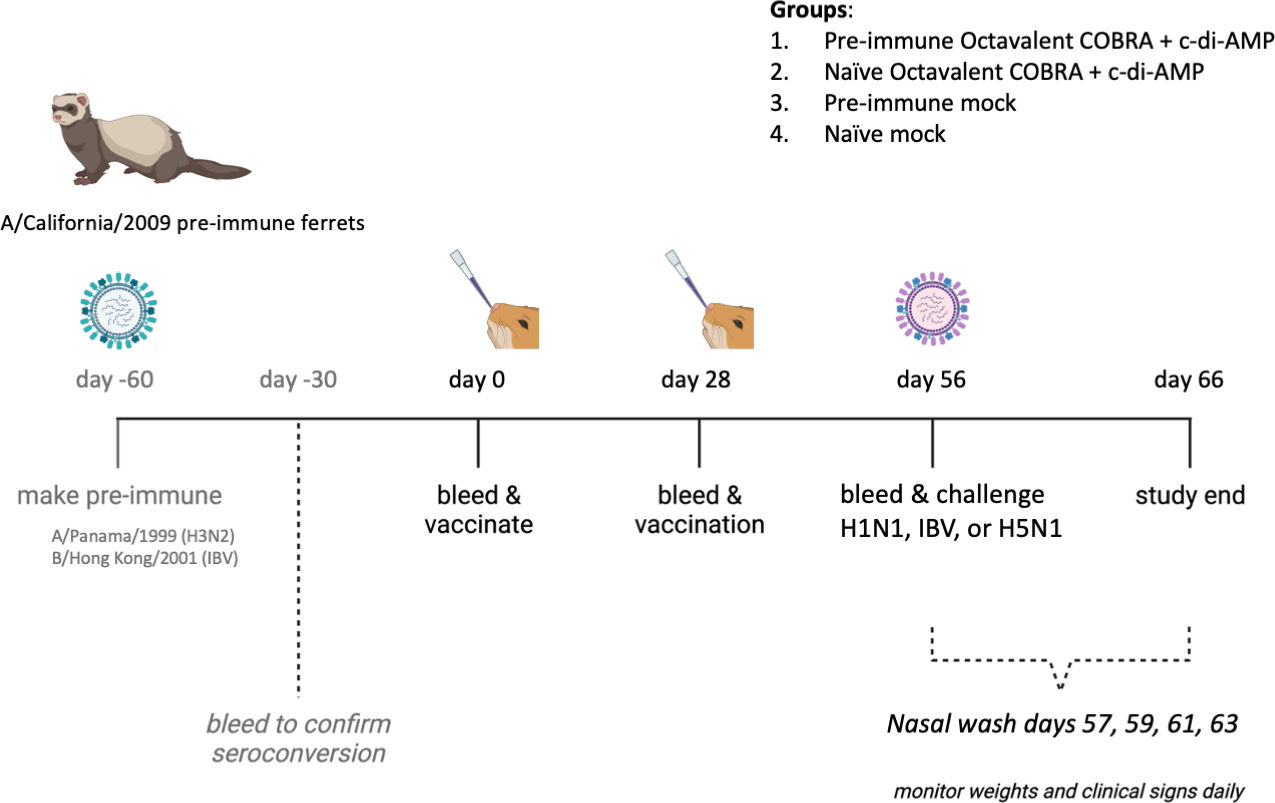
Diagram of ferret vaccine study. For the pre-immune vaccine groups, ferrets that had been exposed to A/California/2009 3 months previously were intranasally infected with A/Panama/1999 and B/Hong Kong/2001 60 days prior to vaccination. On days 0 and 28, pre-immune and naïve groups were vaccinated intranasally with c-di-AMP as adjuvant. Sera were collected on days 0, 28, and 56. Ferrets were challenged with A/Brisbane/02/2018 (H1N1), B/Washington/02/2019 (IBV), or A/Vietnam/1203/2004 (H5N1) on day 56. Ferrets were weighed and monitored for clinical signs daily. Nasal washes were taken on days 57, 59, 61, and 63.

The octavalent formulation was composed of COBRA HA and NA recombinant proteins previously tested in our lab [Table 1; Fig. S1 (15–20, 28)]. Most people have pre-existing immune responses to seasonal influenza viruses. The octavalent COBRA-based vaccine formulation was tested in ferrets that had previously been infected with the historical seasonal influenza viruses CA/09 (H1N1), Pan/99 (H3N2), and B/HK/01 (IBV) (Fig. 1). These pre-immune ferrets were vaccinated intranasally with the eight recombinant proteins (15 µg per antigen) and formulated with c-di-AMP as an adjuvant. A separate group of immunologically naive ferrets was vaccinated, as well as a group of ferrets that were mock vaccinated with adjuvant only as controls (Fig. 1).

**TABLE 1:** COBRA vaccine components^*a*^

	COBRA	Protein	Description
Seasonal	Y4	H1 HA	Based on sequences from 6,232 human H1N1 viruses collected from 1 May 2013 to 30 April 2019
	NG3	H3 HA	Based on sequences from 54,041 human H3N2 viruses collected between 1 May 2008 to 30 September 2019
	N1I	N1 NA	Based on sequences from 4,891 avian viruses collected in 2000–2015, 3,515 swine viruses collected in 1990–2015, and 9,976 human viruses collected in 2001–2014
	N2A	N2 NA	Based on sequences from human viruses collected between 1957 and 2019 and swine viruses collected between 1977 and 2019
	BC3	IBV HA	Based on sequences from 217 human viruses collected between 1999 and 2011
Pandemic	Z1	H2 HA	Based on 382 sequences from avian viruses collected in 1960–2020, human viruses collected in 1957–2020, and swine viruses collected in 1970–2020
	IAN8	H5 HA	Based on 4,524 sequences from avian and human viruses collected from 2011 to 2017
	Q6	H7 HA	Based on 3,633 sequences from avian and human viruses collected from 2000 to 2018

^*a*^ Computationally optimized broadly reactive antigen (COBRA) methodology was used to design HA and NA immunogens for the octavalent vaccine formulation, consisting of both seasonal and pandemic components. Individual COBRA immunogen design methods are detailed.

**Fig 2:**
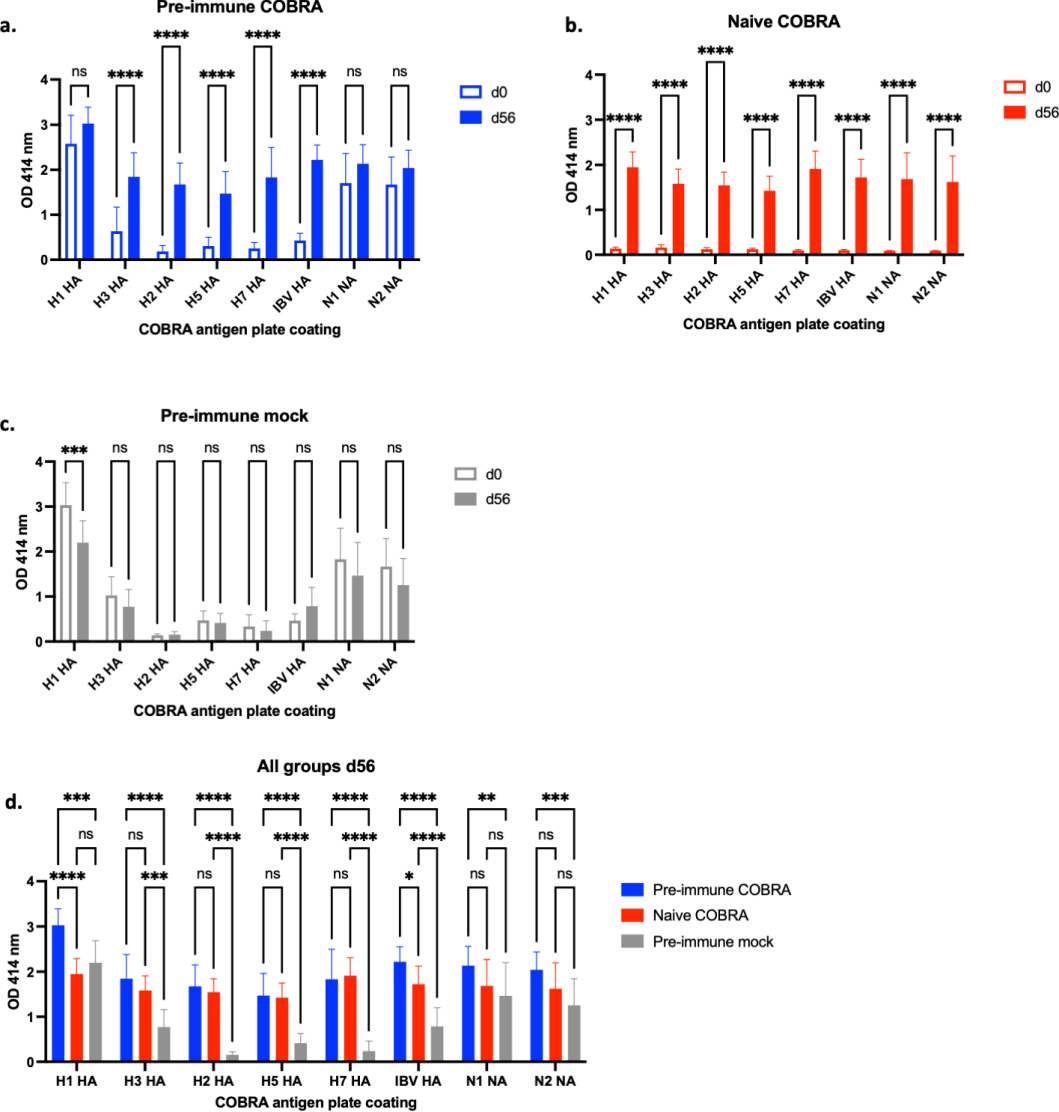
Octavalent COBRA vaccination elicits IgG antibody response in ferrets. Ferrets were vaccinated as described in Fig. 1. Total IgG antibody titers were determined against each of the eight COBRA components, as indicated on the *x*-axis, from sera collected before vaccination (d0, open bars) and after final vaccination (d56, closed bars) from (**a**) pre-immune ferrets given COBRA vaccination, (**b**) naïve ferrets given COBRA vaccination, and (**c**) pre-immune ferrets given mock vaccination. Total IgG antibody titers from d56 sera across all groups were analyzed (**d**). Two-way analysis of variance with multiple comparisons was used to analyze the statistical differences between d0 and d56 ELISA results for each group and d56 ELISA results across all groups by GraphPad Prism version 9 software (GraphPad, San Diego, CA, USA). A *P* value of <0.05 was defined as statistically significant. *P < 0.05, **P < 0.01, ***P < 0.001, ****P < 0.0001. ELISA, enzyme-linked immunosorbent assay; ns, not significant.

To determine if all eight components in the octavalent COBRA vaccine elicited immune responses, anti-HA and anti-NA serum IgG antibody titers were determined from blood samples collected prior to and after vaccination (Fig. 2; Fig. S2). There were no detectable anti-HA or anti-NA antibodies in naive ferrets prior to vaccination (Fig. S2a; Fig. 2b). However, following vaccination, there was a significant rise (*P* > 0.0001) in IgG antibody binding titers to all eight components in the vaccine (Fig. 2b). Ferrets infected with historical influenza viruses had detectable IgG serum antibodies against the H1 HA and both NA proteins 60 days after infection (Fig. 2a and c). There was a significant rise in titers against all vaccine components post-vaccination (Fig. 2a). There was no change in antibody titers in mock vaccinated, pre-immune ferrets, except for a decrease in H1 antibody titer (Fig. 2c). Pre-immune ferrets given COBRA vaccinations had the highest antibody titers, and the naive COBRA vaccinated ferrets had similar or higher antibody titers compared to pre-immune mock ferrets (Fig. 2d)

**Fig 3:**
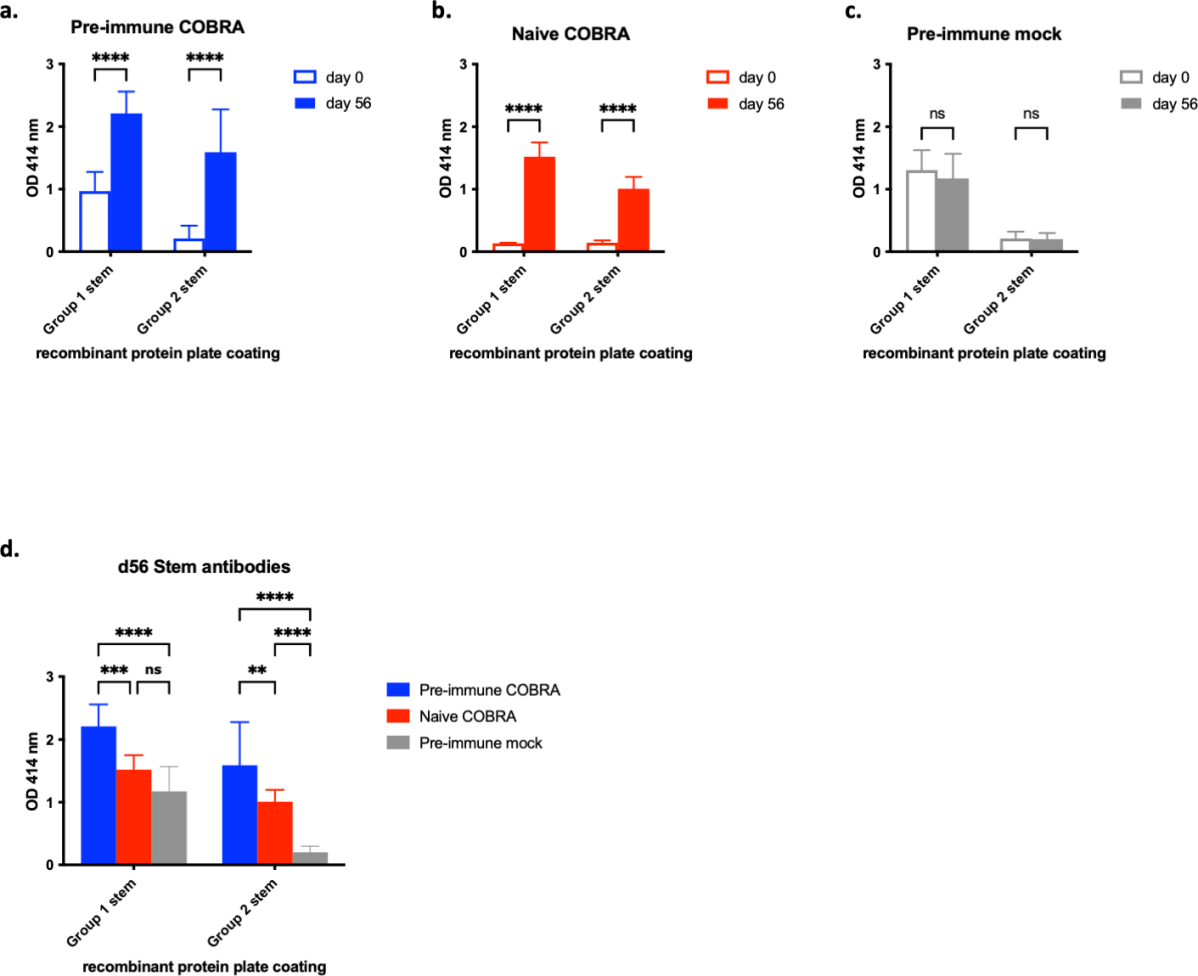
Octavalent COBRA vaccination elicits IgG antibody response against group 1 and group 2 stem proteins. Ferrets were vaccinated as described in Fig. 1. Total IgG antibody titers were determined against group 1 and group 2 stem proteins, as indicated on the *x*-axis, from sera collected before vaccination (d0, open bar) and after final vaccination (d56, closed bar) from (**a**) pre-immune ferrets given COBRA vaccination, (**b**) naïve ferrets given COBRA vaccination, and (**c**) pre-immune ferrets given mock vaccination. Total IgG antibody titers from d56 sera across all groups were analyzed (**d**). Two-way analysis of variance with multiple comparisons was used to analyze the statistical differences between d0 and d56 ELISA results for each group and d56 ELISA results across all groups by GraphPad Prism version 9 software (GraphPad). A *P* value of <0.05 was defined as statistically significant. **P < 0.01, ***P < 0.001, ****P < 0.0001. ns, not significant.

Antibodies elicited against the HA stem were also detected. The HA stem is more conserved than the HA head domain, and anti-stem antibodies are cross-reactive and bind to strains within a phylogenetic group (29, 30). Pre-immune ferrets had strong anti-group 1 stem antibodies, but little or no anti-group 2 stem antibodies following infection (Fig. 3). There was a significant rise in anti-stem antibodies following COBRA-based vaccination (Fig. 3a). Similarly, naive vaccinated ferrets had a significant rise in anti-stem titers against both group 1 and group 2 stem proteins following two vaccinations (Fig. 3b). Pre-immune COBRA vaccinated ferrets significantly had the highest total IgG titers against group 1 and group 2 stem proteins, followed by naive COBRA vaccinated ferrets, then pre-immune mock vaccinated ferrets (Fig. 3d).

**Fig 4:**
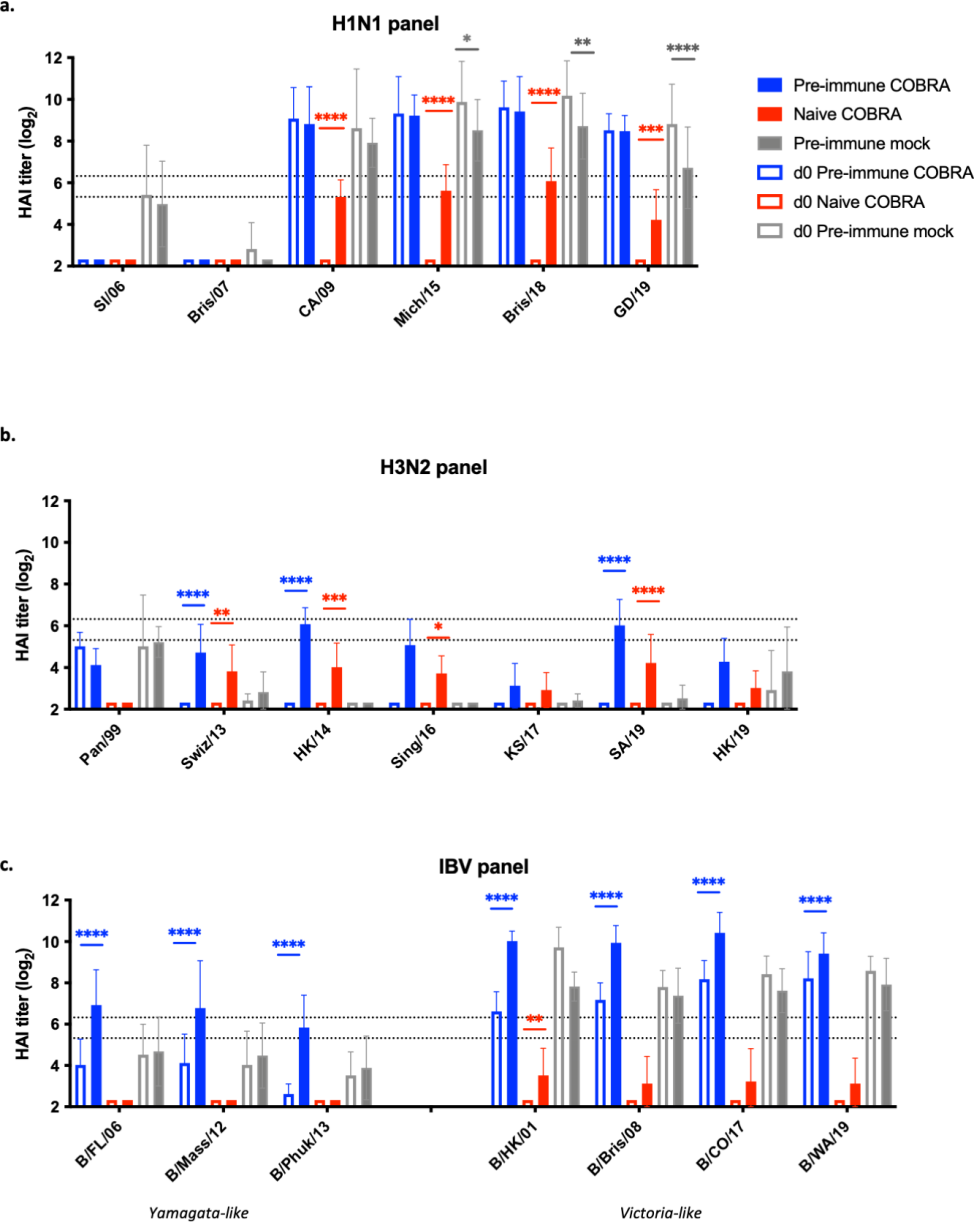
HAI antibody titers for seasonal viruses are increased in octavalent COBRA vaccinated ferrets. Ferrets were vaccinated as described in Fig. 1. Sera were collected before vaccination (open bars) and 4 weeks after the second vaccination (closed bars) for HAI assay against a panel of (**a**) H1N1, (**b**) H3N2, and (**c**) influenza B viruses. The virus strains are listed along the *x*-axis. The *y*-axis indicates the log_2_ HAI titers for each vaccinated group and presents them as absolute mean values ± SEM. The dotted lines indicate HAI titers ranging from 1:40 (lower line) and 1:80 (upper line). Statistical differences between day 0 and day 56 HAI titers for each vaccine group were analyzed using two-way analysis of variance with multiple comparisons by GraphPad Prism version 9 software (GraphPad). A *P* value of <0.05 was defined as statistically significant. *P < 0.05, **P < 0.01, ***P < 0.001, ****P < 0.0001. HAI, hemagglutination inhibition.

Pre-immune ferrets had antibodies with hemagglutination inhibition (HAI) activity against the post-pandemic H1N1 strains in 2009, 2015, 2018, and 2019, prior to vaccination (Fig. 4a). There was little HAI activity against pre-2009 H1N1 viruses. After vaccination, these same ferrets maintained high HAI titers against the post-2009 H1N1 strains. In contrast, pre-immune ferrets that were mock vaccinated had a fourfold decline in HAI activity against 2015 and 2018 H1N1 viruses and an eightfold drop against the 2019 strain following vaccination on day 56 (Fig. 4a). Immunologically naive ferrets had a significant increase in antibodies with HAI activity against post-pandemic strains following vaccination, albeit lower than antisera from pre-immune ferrets (Fig. 4a).

Sera collected from pre-immune ferrets had no HAI activity against the panel of modern H3N2 strains prior to vaccination; they had HAI activity against pre-immune virus Pan/99, as expected (Fig. 4b). After vaccination, pre-immune and naive ferrets had antibodies with significant increases in HAI titers against four out of six H3N2 strains in the panel: Sw/13 (clade 3C.3a), HK/14 (clade 3 c.2a), Sing/16 (clade 3C.2a1), and SA/19 (clade 3C.2a1b/131K). There was no significant increase in HAI titers against KS/17 (clade 3C.3a) or HK/19 (clade 3C.2a1b/137F). Pre-immune COBRA vaccinated ferrets had the highest HAI antibody titers compared to the other groups.

Ferrets infected with B/HK/01 (B/Vic lineage) had antibodies with HAI titers against IBV strains from both lineages; however, the HAI titers were significantly higher against viruses in the B/Vic IBV panel (Fig. 4c). After vaccination, pre-immune ferrets had significantly higher HAI titers (d56) against all strains from both lineages compared to pre-vaccination (d0). Immunologically naive ferrets vaccinated with the same octavalent COBRA vaccine formulation had slight increases in HAI titers against IBV strains from the B/Vic lineage, but not for the B/Yam lineage (Fig. 4c).

**Fig 5:**
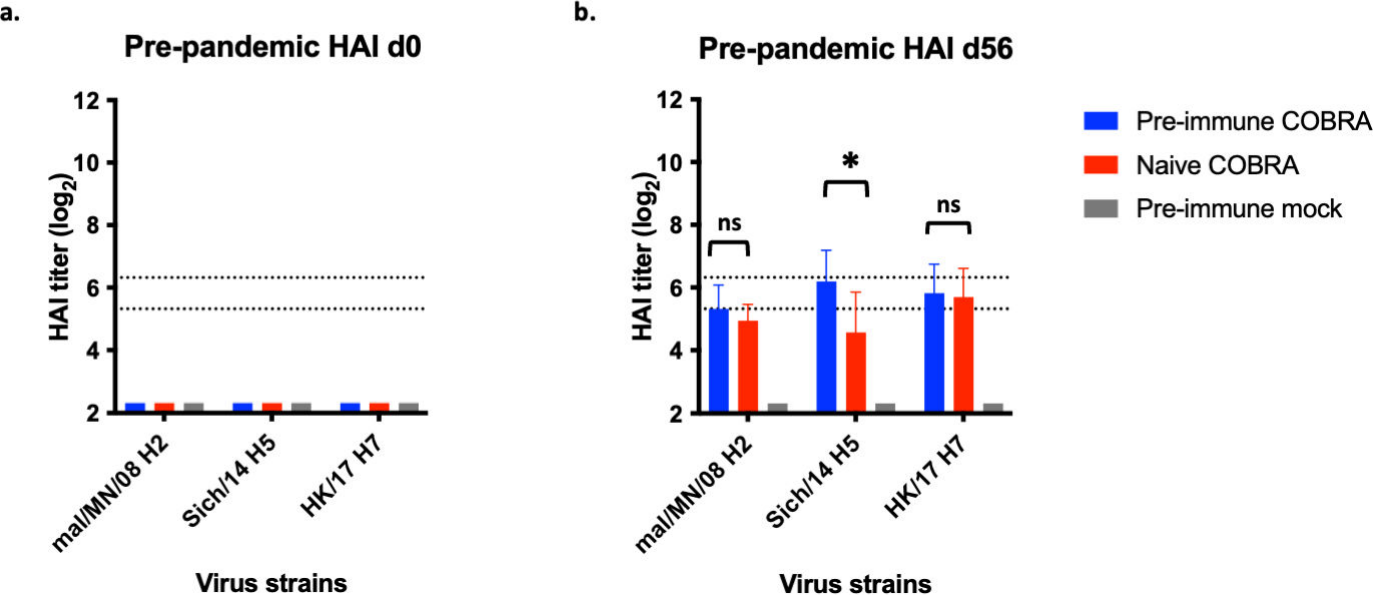
Octavalent COBRA vaccination elicits HAI antibody titers for pre-pandemic viruses in ferrets. Ferrets were vaccinated as described in Fig. 1. Sera were collected before vaccination (**a**) and 4 weeks after the second vaccination (**b**) for HAI assay against a panel of pre-pandemic reassortant viruses. The virus strains are listed along the *x*-axis. The *y*-axis indicates the log_2_ HAI titers for each vaccinated group and presents them as absolute mean values ± SEM. The dotted lines indicate HAI titers ranging from 1:40 (lower line) and 1:80 (upper line). Statistical differences between day 56 HAI titers for each vaccine group were analyzed using unpaired *t*-tests by GraphPad Prism version 9 software (GraphPad). A *P* value of <0.05 was defined as statistically significant. *P < 0.05. ns, not significant.

HAI antibody titers against pre-pandemic viruses were analyzed before and after vaccination (Fig. 5). Prior to vaccination, collected sera had no HAI activity against any influenza strains in the ferrets (Fig. 5a). COBRA HA/NA vaccination elicited antibodies with HAI activity against H2, H5, and H7 in pre-immune and naive ferrets (Fig. 5b). The HAI titers against both the H2 and H7 influenza strains were statistically similar. Ferrets pre-immune to historical H1N1, H3N2, and IBV strains had statistically higher H5 HAI titers in pre-immune animals vaccinated with the octavalent COBRA-adjuvant mix compared to immunologically naive but subsequently COBRA-adjuvant vaccinated ferrets.

**Fig 6:**
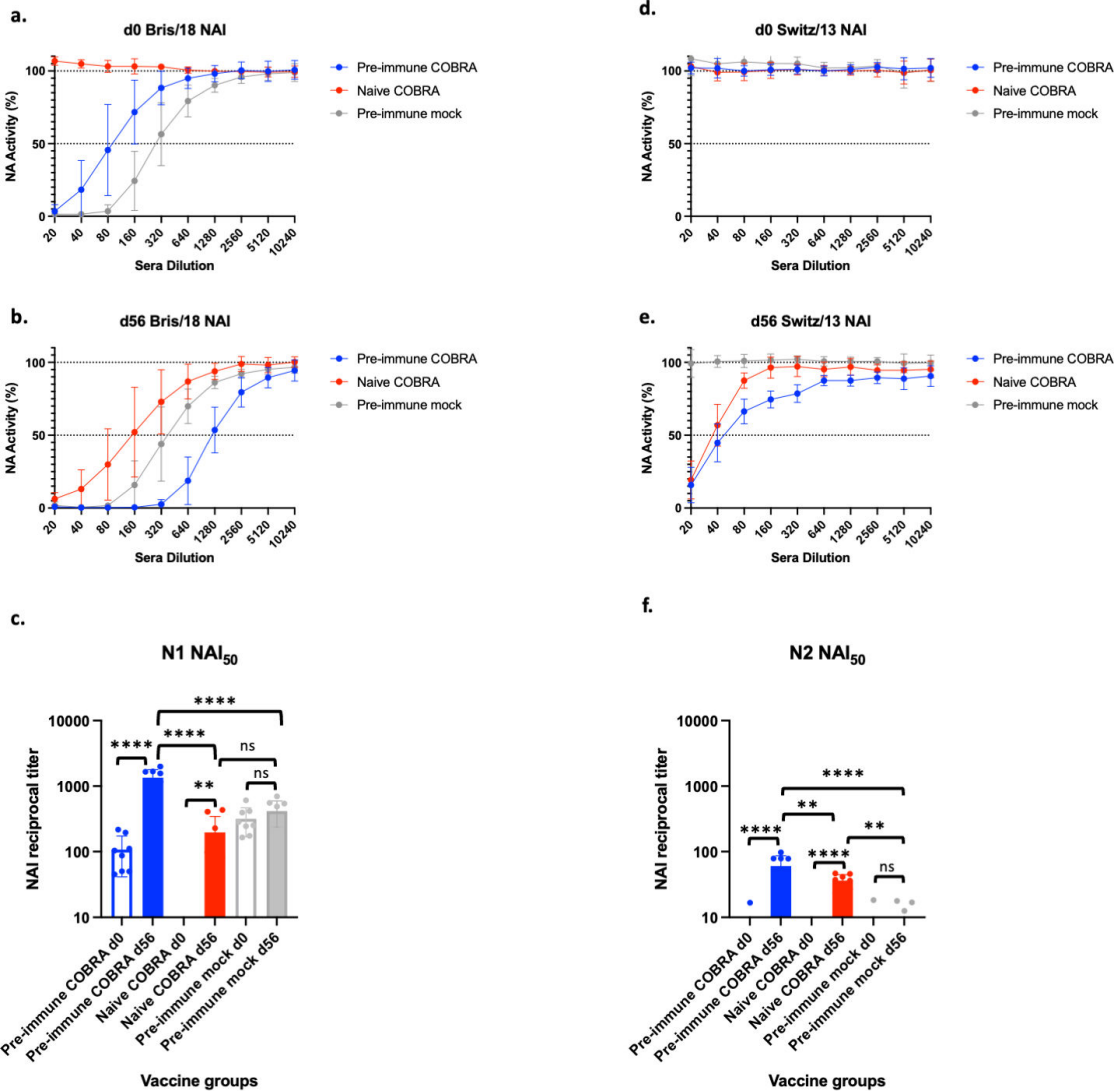
Octavalent COBRA vaccination increases serum NAI antibody titers after vaccination in ferrets. Ferrets were vaccinated as described in Fig. 1. Sera were collected before vaccination (d0) and 4 weeks after the second vaccination (d56) for NAI assay against (**a–c**) N1 and (**d–f**) N2 recombinant proteins. (**c and f**) Statistical differences between NAI_50_ titers were analyzed using one-way analysis of variance with multiple comparisons by GraphPad Prism version 9 software (GraphPad). A *P* value of <0.05 was defined as statistically significant. **P < 0.01, ****P < 0.0001. ns, not significant.

Pre-immune ferrets had pre-existing neuraminidase inhibition (NAI) titers against N1 (Fig. 6a) but no pre-existing titers against N2 prior to vaccination (Fig. 6d). Following vaccination, the NAI titers increased against both the N1 (Fig. 6b) and N2 components (Fig. 6e). Pre-immune ferrets that were vaccinated with the octavalent COBRA formulation had significantly higher NAI_50_ titers at day 56 against the N1 component compared to naïve octavalent COBRA vaccinated ferrets. Both pre-immune and naive ferrets had elevated NAI_50_ titers following vaccination compared pre-immune ferrets that were mock vaccinated (Fig. 6c). Similarly, pre-immune ferrets that were vaccinated with the octavalent COBRA vaccine formulation had the highest NAI_50_ titers against the N2 component, followed by naïve ferrets given octavalent COBRA vaccination, then pre-immune ferrets given mock vaccination (Fig. 6f).

### Octavalent COBRA-based vaccination protected ferrets against influenza virus infection

**Fig 7:**
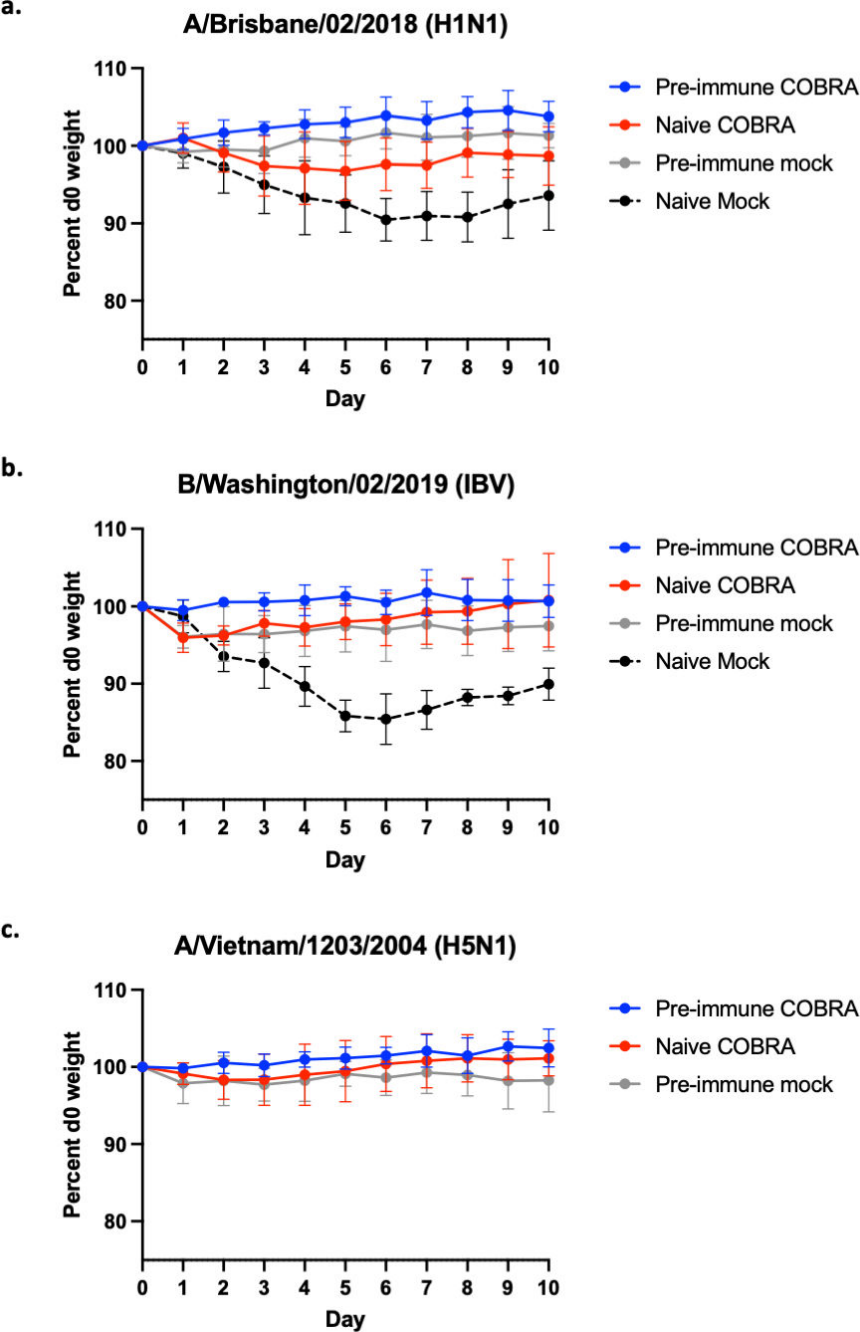
Octavalent COBRA vaccination or pre-existing immunity protects against seasonal or pandemic virus challenges. Ferrets were vaccinated as described in Fig. 1. Vaccine groups are pre-immune ferrets given octavalent COBRA recombinant HA and NA (blue line), naïve ferrets given octavalent COBRA recombinant HA and NA (red line), pre-immune ferrets given mock vaccination (gray line), or naïve ferrets given mock vaccination (black dashed line). Pre-immune ferrets were previously infected with A/California/2009, A/Panama/1999, and B/Hong Kong/2001. Four weeks after final vaccination, ferrets were intranasally infected with (**a**) A/Brisbane/02/2018 (10^8^ PFU), (**b**) B/Washington/02/2019 (10^7^ PFU), or a lethal dose of (**c**) A/Vietnam/1203/2004 (10^5^ PFU) in a volume of 1 mL. The animals were observed for clinical signs, and their body weight was recorded daily post-infection.

Ferrets were challenged with H1N1, H5N1, or IBV strains to evaluate the breadth of protection of octavalent COBRA vaccine. Following the Bris/18 H1N1 influenza virus infection, both vaccinated immunologically naive and vaccinated pre-immune ferrets had significantly less weight loss than the naive mock vaccinated ferrets (Fig. 7a; Table 2a). Similarly, after B/WA/19 (B/Vic) infection, both pre-immune and immunologically naive ferrets vaccinated with the COBRA-based vaccine had significantly less weight loss than the naive mock vaccinated ferrets (Fig. 7b; Table 2b). All vaccinated naive or pre-immune ferrets survived lethal H5N1 Viet/04 infection (Fig. 7c). Pre-immune COBRA-based vaccinated ferrets had the least weight loss following infection with any of all three infections (Table 2).

**TABLE 2:** Statistical differences of body weight loss between ferret vaccine groups^*a*^

(a) A/Brisbane/02/2018 (H1N1) challenge	d1	d2	d3	d4	d5	d6	d7	d8	d9	d10
Pre-immune COBRA vs naïve COBRA	ns	ns	*	**	**	***	**	**	**	*
Pre-immune COBRA vs pre-immune mock	ns	ns	ns	ns	ns	ns	ns	ns	ns	ns
Pre-immune COBRA vs naïve mock	ns	ns	**	****	****	****	****	****	****	****
Naïve COBRA vs pre-immune mock	ns	ns	ns	ns	ns	ns	ns	ns	ns	ns
Naïve COBRA vs naïve mock	ns	ns	ns	ns	ns	***	**	****	**	*
Pre-immune mock vs naïve mock	ns	ns	ns	***	****	****	****	****	****	***

(b) B/Washington/02/2019 (IBV) challenge	d1	d2	d3	d4	d5	d6	d7	d8	d9	d10
Pre-immune COBRA vs naïve COBRA	ns	ns	ns	ns	ns	ns	ns	ns	ns	ns
Pre-immune COBRA vs pre-immune mock	ns	*	*	ns	ns	ns	*	ns	ns	ns
Pre-immune COBRA vs naïve mock	ns	***	****	****	****	****	****	****	****	****
Naïve COBRA vs pre-immune mock	ns	ns	ns	ns	ns	ns	ns	ns	ns	ns
Naïve COBRA vs naïve mock	ns	ns	*	***	****	****	****	****	****	****
Pre-immune mock vs naïve mock	ns	ns	ns	***	****	****	****	****	****	***

(c) A/Vietnam/1203/2004 (H5N1) challenge	d1	d2	d3	d4	d5	d6	d7	d8	d9	d10
Pre-immune COBRA vs naïve COBRA	ns	ns	ns	ns	ns	ns	ns	ns	ns	ns
Pre-immune COBRA vs pre-immune mock	ns	ns	ns	ns	ns	ns	ns	ns	*	*
Naïve COBRA vs pre-immune mock	ns	ns	ns	ns	ns	ns	ns	ns	ns	ns

^*a*^ Body weight loss values from ferrets challenged with (a) A/Brisbane/02/2018, (b) B/Washington/02/2019, or (c) A/Vietnam/1203/2004 were analyzed for statistical differences for between each group each day by two-way analysis of variance with multiple comparisons by GraphPad Prism version 9 software (GraphPad). A *P* value of less than 0.05 was defined as statistically significant. *P < 0.05, **P < 0.01, ***P < 0.001, ****P < 0.0001. ns, not significant.

**Fig 8:**
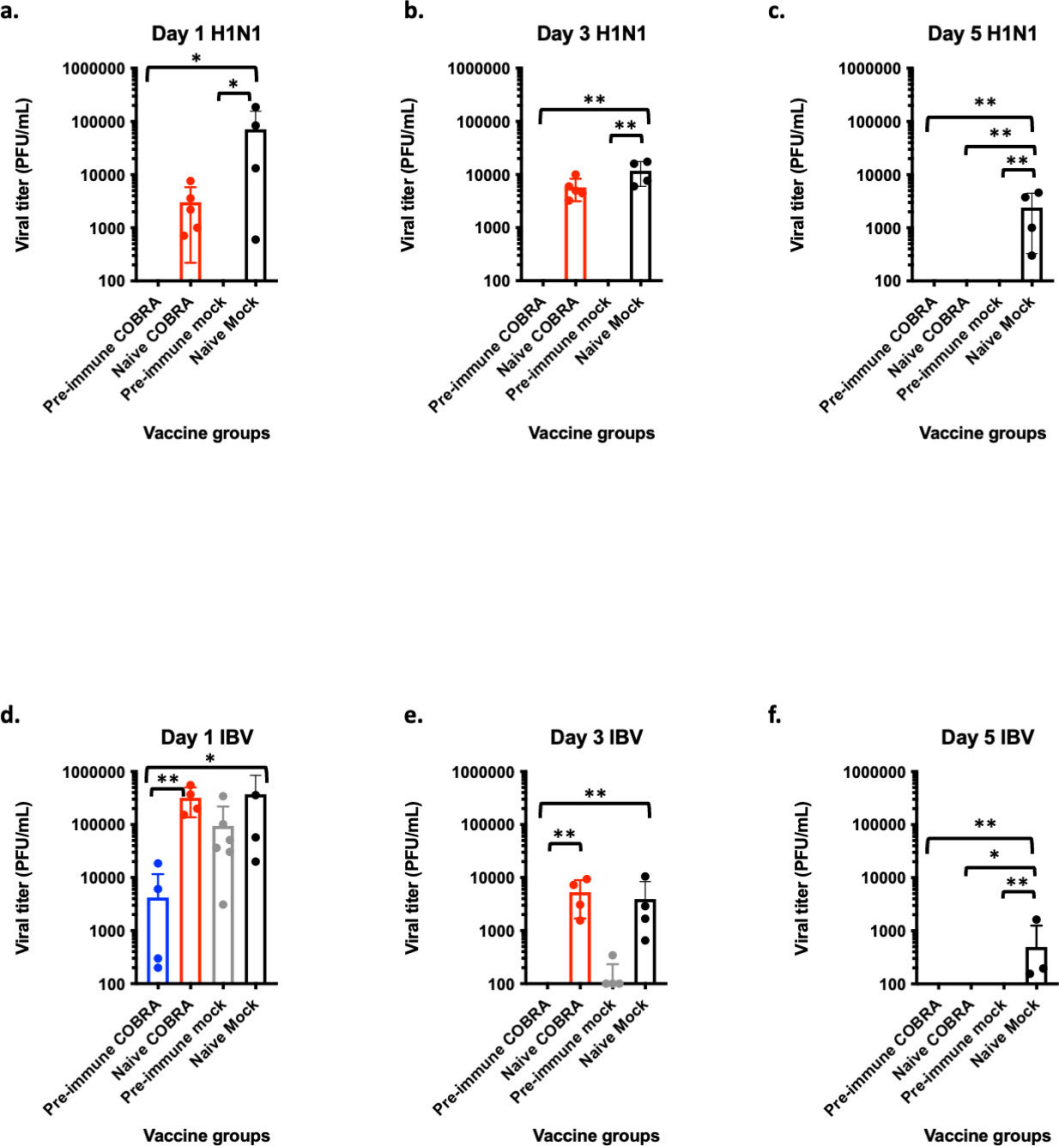
Viral titers in the upper respiratory tract of ferrets after seasonal infection are reduced after octavalent COBRA vaccination. Ferrets were vaccinated as described in Fig. 1. Vaccine groups are indicated on the *x*-axis. Four weeks after the second vaccination, the groups were challenged with H1N1 A/Brisbane/02/2018 (top row, a–c) or IBV B/Washington/02/2019 (bottom row, d–f). Each figure represents viral titers from nasal washes taken on the indicated day post-infection—day 1, day 3, or day—as quantified by viral plaque assay. Viral titers in nasal washes are presented as PFU per milliliter as shown on the *y*-axis. Each dot represents an individual ferret. Statistical differences were analyzed by one-way analysis of variance with multiple comparisons by GraphPad Prism version 9 software (GraphPad). A *P* value of less than 0.05 was defined as statistically significant. *P < 0.05, **P < 0.01.

Following Bris/18 H1N1 challenge, pre-immune ferrets had significantly less detectable virus in their nasal washes compared to naive mock vaccinated ferrets (Fig. 8a through c). Immunologically naive COBRA vaccinated ferrets had significantly less detectable virus in the nasal washes on 5 d.p.i. compared to naive mock vaccinated ferrets (Fig. 8c). Following B/WA/19 (B/Vic) challenge, pre-immune COBRA vaccinated ferrets had significantly lower viral nasal wash titers compared to naive mock vaccinated ferrets (Fig. 8d through f). By 5 d.p.i., both pre-immune and COBRA vaccinated ferrets had undetectable viral titers (Fig. 8f).

## DISCUSSION

In this study, a COBRA-based octavalent influenza vaccine was formulated with a cyclic dinucleotide (CDN) that have been extensively researched as novel molecular adjuvant. This secondary signaling molecule activates the STING protein, resulting in type I IFN and TNF-α (interferon and tumor necrosis factor-alpha) production (31, 32). The c-di-AMP is a CDN capable of stimulating both systemic and mucosal immune responses (31). Intranasal vaccination with c-di-AMP provides cellular and humoral immunity with balanced Th1/Th2/Th17 responses (27) and broad breadth of antigen-specific IgG subclasses (24).

Ferrets administered intranasally with the octavalent COBRA-based vaccine formulated with c-di-AMP adjuvant elicited a broad breadth of immune response against seasonal and pre-pandemic influenza viruses isolated over multiple past seasons. Ferrets are the gold standard for influenza virus infection since they are susceptible to human influenza virus strains and display similar pathological and immunological responses to humans (33, 34). Ferrets can also recapitulate the immune imprinting that humans experience following influenza virus infections, which often affect immune responses against subsequent viral infections and flu vaccinations (35, 36). Examining how the back-boosting phenomenon can influence memory immune responses and generate a cross-reactive response against new strains is critical for development of a universal influenza vaccine. For this study, ferrets that had been previously exposed to CA/09 were used. Most humans have pre-existing antibodies against H3N2 and IBV as well, since these three subtypes make up commercial influenza vaccines. The CA/09 pre-immune ferrets were infected with Pan/99 (H3N2) and B/HK/01 (IBV) to mimic potential human immune repertoires.

Octavalent COBRA-based vaccination elicited broad breadth of HAI activity against all seasonal subtypes tested in this study (Fig. 4). An HAI titer of 1:40 is the minimum standard accepted titer that correlates with protection against seasonal influenza viruses following flu vaccination (37). Pre-immune ferrets administered COBRA-based vaccination had HAI titers greater than 1:40 against all circulating strains in our H1N1 and IBV panels. However, HAI titers against H3N2 viruses were lower due to the absence of pre-existing immunity to these strains. Ferrets were first infected with CA/09 then Pan/99 and B/HK/01. Thus, the highest HAI titers were detected against H1N1, which may be due to initial infection with this viral subtype. Additional studies would have to be performed to determine if this an accurate assessment; the order of pre-immune infections would be interchanged, with H3N2 or IBV being primary infections. This will elucidate the effect of immune imprinting to the effectiveness of the vaccine. Naive ferrets had antibody responses following vaccination, though HAI titers were quite low against the H3N2 or IBV viruses. This may be due to the immunodominance of H1 HA (17). In naive ferrets, an additional vaccination may be necessary to increase HAI titers against H3N2 and IBV viruses to the minimal protective level. Naive mice administered three vaccinations of COBRA H3 HA protein achieved titers of ≥1:40 (17). Alternatively, an increased dosage for the less immunogenic components can be included in the formulation. We have recently developed a more broadly reactive COBRA IBV HA, BC2, that can be tested in a multivalent formulation in the future (19).

Although there are no universally accepted correlates of protection for pre-pandemic HAI titers (2), COBRA vaccination elicited antibodies with HAI activity against H2, H5, and H7 viruses. The pre-pandemic HAI titers were not as robust as seasonal HAI titers. Pre-immune but COBRA vaccinated ferrets had the highest HAI titers (Fig. 5). Similarly, there are no universally accepted correlate of protection for NAI (38), but COBRA HA/NA vaccination elicited NAI titers for both N1 and N2, with the highest NAI titers in pre-immune ferrets that were vaccinated with COBRA HA/NA proteins (Fig. 6). N2 NA NAI titers were lower than N1 NA NAI titers for all groups, despite enzyme-linked immunosorbent assay (ELISA) titers being similar (Fig. 2). N2 NA NAI titer is consistently lower than N1 NA NAI in COBRA NA vaccinated animals. This may be due to low enzymatic activity of N2 NA compared to N1 NA. The magnitude of NAI titer may not correlate to protection, only a minimum NAI antibody titer as a threshold of protection (39). This will need to be examined in future studies.

COBRA HA/NA vaccination protected ferrets against weight loss and reduced viral shedding after seasonal virus challenges (Bris/18 or B/WA/19) compared to mock groups. Naive vaccinated ferrets were protected against lethal H5N1 infection (Viet/04). In our previous studies, the lethal dose of the H5N1 VN/04 influenza was determined in naive ferrets (39, 40), where all animals succumbed to disease between days 5 and 10 post-infection. For this study, the same virus lot and lethal dose were used. Pre-immune mock ferrets were also protected, despite having no H5-specific HAI titers. Our lab and others have shown that pre-existing immunity to H1N1 can confer protection against H5N1 infection (39–41). Anti-stem HA antibodies can aid in neutralization by inhibiting fusion (42) or mediating anti-viral activity via Fc-receptor mediated antibody-dependent cellular cytotoxicity (43). HA stem-based antibodies are more conserved and cross-reactive within an influenza group compared to HA head-binding antibodies (44, 45). COBRA vaccination elicited stem antibodies against both group 1 and group 2 viruses (Fig. 3). Future studies will examine separate protective roles of HA and NA COBRA immunogens and analyze the mucosal immune response to intranasal vaccination with c-di-AMP by collecting samples from the respiratory tract.

## MATERIALS AND METHODS

### Influenza viruses

Influenza viruses were obtained through either the Influenza Reagents Resource, BEI Resources, the Centers for Disease Control (CDC), or provided by Virapur (San Diego, CA, USA). Viruses were passaged once in the same growth conditions as they were received, either embryonated chicken eggs or semi-confluent Madin-Darby canine kidney (MDCK) cell cultures as per the instructions provided by the World Health Organization (WHO) (46).

Virus lots were titered with 0.8% turkey erythrocytes and made into aliquots for single-use applications. H3N2 virus lots were titered with 0.75% guinea pig erythrocytes in the presence of 20-nM oseltamivir and made into aliquots for single-use applications. H5 virus lots were tittered with 1% horse erythrocytes and made into aliquots for single-use applications.

H1N1 influenza viruses used in this study include A/Solomon Islands/03/2006 (SI/06), A/Brisbane/59/2007 (Bris/07), A/California/07/2009 (CA/09), A/Michigan/45/2015 (Mich/15), A/Brisbane/02/2018 (Bris/18), and A/Guangdong-Maonan/SWL1536/2019 (GD/19).

H3N2 influenza viruses used in this study include A/Panama/2007/1999 (Pan/99); A/Switzerland/9715293/2013 (Switz/13, clade 3C.3a); A/Hong Kong/4801/2014 (HK/14, clade 3C.2a); A/Singapore/IFNIMH-16–0019/2016 (Sing/16, clade 3C.2a1); A/Kansas/14/2017 (KS/17, clade 3C.3a); A/South Australia/34/2019 (SA/19, clade 3C.2a1b/131K); and A/Hong Kong/2671/2019 (HK/19, clade 3C.2a1b/137F).

IBVs used in this study include the following: for Yamagata-like lineages, B/Florida/04/2006 (B/FL/06), B/Massachusetts/02/2012 (B/Mass/12), and B/Phuket/3073/2013 (B/Phuk/13); and for Victoria-like lineages, B/Hong Kong/330/2001 (B/HK/01), B/Brisbane/60/2008 (B/Bris/08), B/Colorado/06/2017 (B/CO/07), and B/Washington/02/2019 (B/WA/19). The IBV viruses used for HAI assays were first ether-extracted to increase HA activity (47).

Pre-pandemic viruses used for HAI were PR8 reassortant viruses: A/mallard/Minnesota/AI08-3437/2008 (mal/MN/08, H2N3), A/Sichuan/26221/2014 (Sich/14, H5N6), and A/Hong Kong/127/2017 (HK/17, H7N9).

The H5N1 virus used for pandemic challenge was A/Vietnam/1203/2004 (Vn/04, H5N1).

### Vaccine design and production

COBRA methodology was used to produce influenza HA and NA recombinant proteins representing seasonal and pandemic subtypes. The design and production of these COBRA antigens have been previously published by our lab (Table 1). Briefly, full-length WT HA or NA sequences were downloaded from Global Initiative on Sharing Avian Influenza Data for each subtype. The sequences were aligned, and primary consensus sequences were created from clusters of WT sequences based on percent similarity. Secondary consensus sequences were created from the primary sequences; this multi-layered consensus building way continued until a final COBRA sequence was obtained (Fig. S1).

The production of recombinant COBRA HA and NA proteins has been previously described (48). Briefly, each COBRA HA or NA nucleotide sequence was cloned into a pcDNA3.1+ plasmid vector, being truncated by removing the transmembrane domain and replacing with a T4 fold-on domain, an AviTag, and a 6× His tag for purification by immobilized metal affinity chromatography. The plasmid was transfected into human embryonic kidney 293T suspension cells for protein expression. Protein concentrations of the purified soluble proteins were determined by bicinchoninic acid assay (BCA) according to the manufacturer’s instructions (Thermo Fisher Scientific, Waltham, MA, USA).

### Viral infection and vaccination of ferrets

Fitch ferrets (*Mustela putorius furo*, female, 6–12 months of age), negative for antibodies to circulating influenza A (H1N1 and H3N2) and influenza B viruses, were de-scented and purchased from Triple F Farms (Sayre, PA, USA). Ferrets were pair housed in stainless steel cages (Shor-line, Kansas City, KS, USA) containing Sani-Chips laboratory animal bedding (P.J. Murphy Forest Products, Montville, NJ, USA). Ferrets were provided with Teklad Global Ferret Diet (Harlan Teklad, Madison, WI, USA) and fresh water *ad libitum*. Prior to bleeds, vaccinations, infections, and nasal washes, ferrets were anesthetized with vaporized isoflurane.

Ferrets were randomly divided into vaccine groups, and each vaccine group had three different infection groups (*n* = 4–6). The groups were pre-immune ferrets given octavalent COBRA vaccine, naive ferrets given octavalent COBRA vaccine, pre-immune ferrets given mock vaccine, and naive ferrets given mock vaccine. For the pre-immune groups, ferrets that had been previously exposed to CA/09 prior to this study were infected with Pan/99 and B/HK/01 [10^6^ plaque-forming unit (PFU) each, mixed in 1-mL total volume] 60 days prior to vaccination. Ferrets were vaccinated intranasally (i.n.) with octavalent formulation of COBRA HA and NA recombinant proteins (Y4, Z1, NG3, IAN8, Q6, BC3, N1I, and N2A). The vaccines contained 15 µg of each antigen and were formulated with 50-µg c-di-AMP as adjuvant (27). Ferrets were boosted 28 days after initial vaccination. Blood was harvested from all anesthetized ferrets via the anterior vena cava prior to vaccination and at days 28 and 56 post-initial vaccination. Serum was transferred to a centrifuge tube and centrifuged at 2,500 rpm. Clarified serum was removed and frozen at −20°C. Ferrets vaccinated with placebo consisted of phosphate-buffered saline (PBS), pH 7.4, formulated with 50 µg of c-di-AMP. All vaccines and placebo were stored in a refrigerator at a temperature between 2°C and 8°C until use.

On day 56 post-vaccination, ferrets were challenged i.n. with H1N1 Bris/18 (10^8^ PFU), IBV B/WA/19 (10^7^ PFU), or a lethal dose of H5N1 Vn/04 (10^5^ PFU, BSL3 select agent) viruses in a volume of 1 mL (*n* = 5 per vaccine group per challenge). Ferrets were monitored daily for weight loss, disease signs, and death for 10 days after infection. Experimental endpoints were defined as >20% wt loss compared to initial body weight. Additionally, dyspnea, lethargy, response to external stimuli, and other respiratory distress were closely monitored for the determination of humane endpoint. Nasal washes were performed by instilling 3 mL of PBS into the nares of anesthetized ferrets on days 1, 3, 5, and 7 post-infection. Washes were collected and stored at −80°C until use.

### ELISA

ELISA was used to assess antibody reactivity before and after ferret vaccination against different HA and NA antigens and was performed as previously described (49). Briefly, Immulon 4HBX plates (Thermo Fisher Scientific) were coated at 4°C overnight with 50 µL/well solution of carbonate buffer (pH 9.4) containing 1 µg/mL of rHA or recombinant NA (rNA), or 5 µg/mL bovine serum albumin (BSA) as a negative control. Plates were blocked with ELISA blocking buffer in a volume of 200 µL/well for 1 h at 37°C. Serum samples were serially diluted threefold in blocking buffer starting from a dilution of 1:100 and then were added into HA protein-coated plates. After incubation at 4°C overnight, plates were washed five times in washing buffer (0.05% Tween-20 in PBS). Goat anti-ferret IgG H&L HRP (100 µL; Abcam, Boston, MA, USA) diluted at 1:4,000 in blocking buffer was added, and the plates were incubated for 1 h at 37°C. The plates were washed five times in washing buffer. Fifty microliters of ABTS substrate (VWR, Radnor, PA, USA) was added into each well and further incubated at 37°C for 15–20 min. Colorimetric conversion was terminated by adding 50 µL of 1% SDS into each well. The optical density (OD) values (OD414) were measured by a spectrophotometer (PowerWave XS BioTek; Agilent Technologies, Santa Clara, CA, USA) at 414 nm.

### HAI assay

The HAI assay was used to assess functional antibodies to the HA that are able to inhibit agglutination of turkey erythrocytes for H1N1, H2N3, H7N9, and IBV viruses; horse erythrocytes for H5N6 virus; and guinea pig erythrocytes for H3N2 viruses. Guinea pig red blood cells (GPRBCs) are frequently used to characterize contemporary A (H3N2) influenza strains that have developed a preferential binding to α2,6-linked sialic acid receptor (50). The protocols were adapted from the WHO lab influenza surveillance manual (51). To inactivate non-specific inhibitors, sera samples were treated with receptor-destroying enzyme (RDE) (Denka Seiken, Co., Japan) prior to being tested. Briefly, three parts of RDE were added to one part of sera and incubated overnight at 37°C. RDE was inactivated by incubation at 56°C for 30 min.

RDE-treated sera were diluted in a series of twofold serial dilutions in v-bottom microtiter plates. An equal volume of virus, adjusted to approximately 8 hemagglutination units (HAU)/50 µL, was added to each well. The plates were covered and incubated at room temperature (RT) for 20 min, and then 0.8% turkey red blood cells (TRBCs) or 1% horse red blood cells (HRBCs) (Lampire Biologicals, Pipersville, PA, USA) in PBS were added. Prior to use, the red blood cells (RBCs) were washed twice with PBS, stored at 4°C, and used within 24 h of preparation. The plates were mixed by gentle agitation and covered, and the RBCs were allowed to settle at room temperature for 30 min for TRBC and 1 h for HRBCs. The HAI titer was determined by the reciprocal dilution of the last well that contained non-agglutinated RBCs. Positive and negative serum controls were included for each plate.

Similarly, for H3N2 HAI, RDE-treated sera were diluted in a series of twofold serial dilutions in v-bottom microtiter plates. An equal volume of each H3N2 virus, adjusted to approximately 8 HAU/50 µL in the presence of 20-nM oseltamivir carboxylate, was added to each well. The plates were covered and incubated at room temperature for 30 min, and then 0.75% GPRBCs (Lampire Biologicals) in PBS were added. Prior to use, the GPRBCs were washed twice with PBS, stored at 4°C, and used within 24 h of preparation. The plates were mixed by gentle agitation and covered, and the GPRBCs were allowed to settle for 1 h at room temperature. The HAI titer was determined by the reciprocal dilution of the last well that contained non-agglutinated RBCs. Positive and negative serum controls were included for each plate.

All animals were negative (HAI ≤ 1:10) for pre-existing antibodies to human influenza viruses prior to infection or vaccination, and for this study, a positive HAI reaction, or “sero-protection,” is defined as an HAI titer of ≥1:40, and “seroconversion” refers to a fourfold increase in titer compared to baseline, as per the WHO and European Committee for Medicinal Products to evaluate influenza vaccines (52). Ferrets made pre-immune prior to vaccination all had HAI titers of ≥1:40.

### Enzyme-linked lectin assay

Enzyme-linked lectin assay (ELLA) was used to assess the NAI activity of polyclonal ferret sera before and after vaccination. First, rNA proteins representing wild-type strains were generated in our lab as an alternative to using whole virus. This was done to prevent HA binding antibodies in the sera from inhibiting NA activity via steric hindrance (53). Bris/18 (N1) and Switz/13 (N2) soluble tetrameric NA proteins were produced by transient transfections, as previously described (48): NA nucleotide sequences were cloned into a pcDNA3.3-TOPO vector (Thermo Fisher Scientific) and transiently transfected into EXPI293F cells (Thermo Fisher Scientific). rNAs were purified by immobilized metal affinity chromatography, and protein concentrations were determined by BCA. NA activity was determined by ELLA, as previously described (54). Briefly, high-affinity Immunoblot 4HBX 96-well flat bottom plates (Thermo Fisher Scientific) were coated with 100 µL of 25-µL/mL fetuin (Sigma-Aldrich, St. Louis, MO, USA) in coating buffer (Seracare Life Sciences, Milford, MA, USA) overnight at 4°C. Plates were washed 3× in wash buffer (0.05% Tween-20 in PBS), and serial dilutions of rNA in sample diluent (Dulbecco’s phosphate-buffered saline [DPBS] containing 0.133-g/L CaCl_2_ and 0.1-g/L MgCl_2_ + 1% BSA + 0.5% Tween-20) were added and incubated for 17 h at 37°C. Plates were then washed 6×. Peanut agglutinin-HRPO (PNA-HRPO, Sigma-Aldrich) was diluted 1,000-fold in conjugant diluent (DPBS + 0.1% BSA), and 100 µL was added to the plates and incubated for 2 h at RT. Plates were washed 3×, and 100 µL of o-phenylenediamine dihydrochloride in phosphate-citrate buffer (Sigma-Aldrich) was added for 10 min at RT. One hundred microliters of 1-N sulfuric acid was added to stop the reaction. Absorbance was read at 490 nm with a spectrophotometer (Epoch 2, Agilent Technologies). The concentration of rNA to use in the NAI assay (90–95% NA activity) was determined by linear regression analysis using GraphPad Prism version 9 software (GraphPad, San Diego, CA, USA).

For the NAI assay, ferret sera were first treated to inactivate non-specific neuraminidase inhibition by inherent serum immunoglobulins by heat inactivation (55). After inactivation, 50-µL twofold serial dilutions of ferret sera in the sample diluent were combined with 50-µL rNA antigen in the optimal concentration determined above. The mixture was placed on 96-well fetuin-coated plates (washed 3× in washing buffer prior to addition) and underwent the rest of the ELLA procedure as described above: 17-h incubation, followed by addition of PNA-HRPO, OPD substrate, and sulfuric acid stop solution. Plates were read at 490 nm on the spectrophotometer. NA percent activity was determined as (serum absorbance − mean background absorbance) ÷ (mean virus-only absorbance − mean background absorbance) × 100. Non-linear regression analysis was used to determine 50% NAI titer on GraphPad Prism version 9 software.

### Influenza viral plaque assay

MDCK cells (Sigma-Aldrich) were seeded into each well of a six-well plate at a concentration of 1 × 10^6^ cells/well 1 day prior to performing the plaque assay. On the day of the assay, nasal wash samples were thawed on ice and serially diluted 10-fold in Dulbecco’s modified Eagle medium (DMEM) supplemented with 1% penicillin-streptomycin (DMEM + P/S) (Thermo Fisher). When MDCK cells reached 90% confluency in each well, the plates were washed 2× with DMEM + P/S and infected with 100 µL of each dilution. The plates were then shaken every 15 min for 1 h. After 1 h of incubation, the supernatant was removed and cells were washed twice with fresh DMEM + P/S. Following the second wash, a solution of 2× minimum essential medium (MEM) and 1.6% agarose (Thermo Fisher) mixed 50:50 vol/vol and supplemented with 1 µg/mL of L-1-tosylamido-2-phenylethyl chloromethyl ketone (TPCK) treated trypsin (Thermo Fisher) was added into each well. Plates were then incubated at 37°C + 5% CO_2_ for 72 h. After 72 h, the gel overlays were removed from each well, and the cells were fixed with 10% buffered formalin for 10 min and stained with 1% crystal violet (Thermo Fisher) for 10  min at room temperature. Plates were then rinsed thoroughly 5× with fresh water to remove excess crystal violet. Plates were allowed to air dry for 24 h, and the viral plaques were enumerated as the reciprocal of each dilution. The viral titers were calculated and presented as PFU per milliliter of nasal wash sample.

### Statistical analysis

Data are presented as absolute mean values ± standard error of the mean. One-way analysis of variance (ANOVA), unpaired multiple *t*-tests, and two-way ANOVA with multiple comparisons were used to analyze the statistical differences between vaccine groups using GraphPad Prism version 9 software (GraphPad). A *P* value of <0.05 was defined as statistically significant (*P < 0.05, **P < 0.01, ***P < 0.001, ****P < 0.0001).

## Data Availability

All data are included in the paper or available upon request.

## SUPPLEMENTAL MATERIAL

Supplemental figures jvi.00354-24-s0001.pdf Fig. S1 and S2.

ASM does not own the copyrights to Supplemental Material that may be linked to, or accessed through, an article. The authors have granted ASM a non-exclusive, world-wide license to publish the Supplemental Material files. Please contact the corresponding author directly for reuse.
