# Supplementary material for: Intranasal administration of octavalent next-generation influenza vaccine elicits protective immune responses against seasonal and pre-pandemic viruses
Source: J Virol. 2024 Aug 22;98(9):e00354-24. doi: 10.1128/jvi.00354-24 (PMC11406897; doi:10.1128/jvi.00354-24)
Supplement: Supplemental figures — Fig. S1 and S2. [file jvi.00354-24-s0001.pdf]

Fig. S1

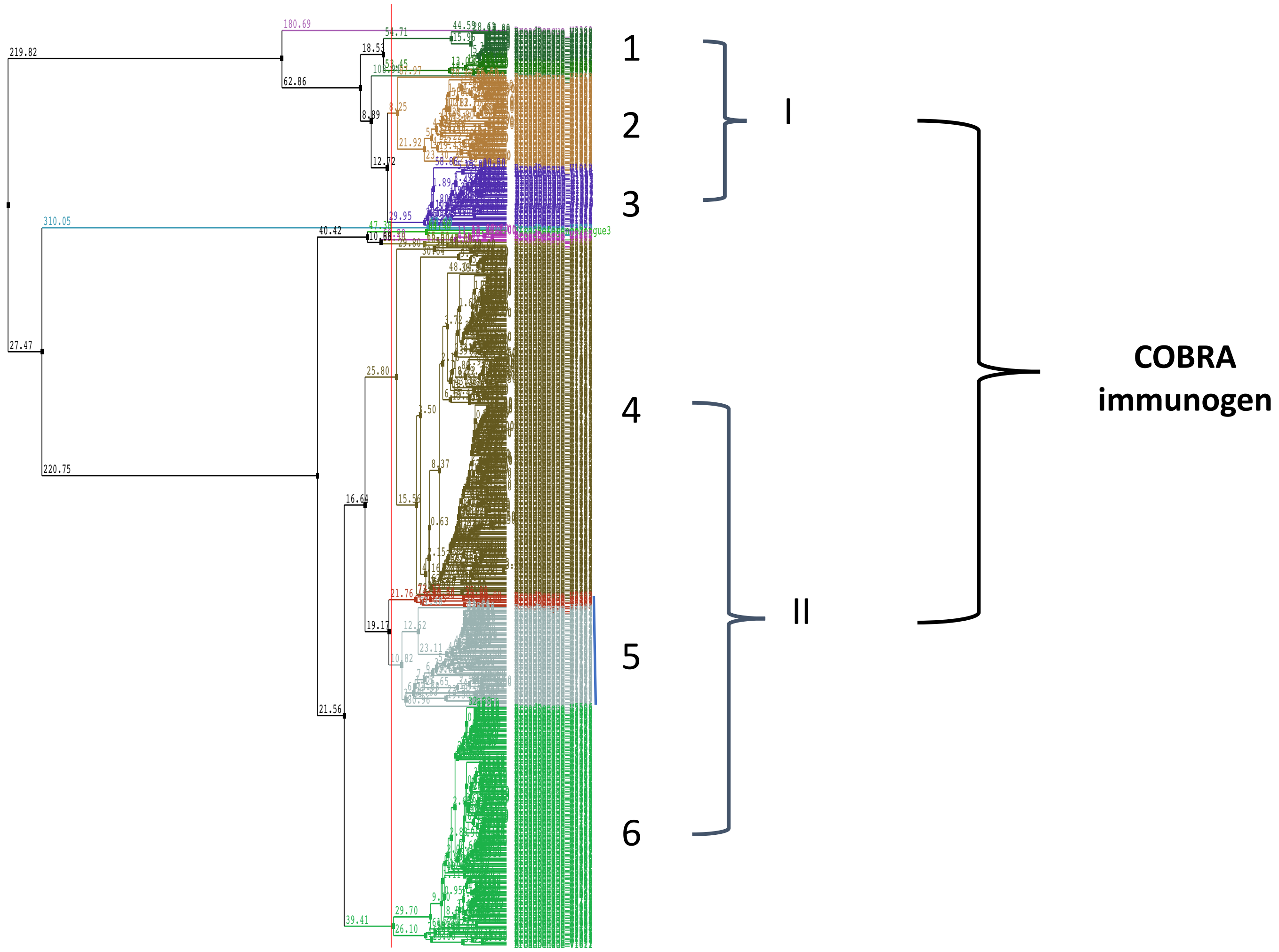

Supplemental figure 1: Schematic for constructing COBRA immunogens. Full length wild-type (WT) HA or NA sequences were downloaded from Global Initiative on Sharing Avian Influenza Data (GISAID) for each subtype. The sequences were aligned and primary consensus sequences (1-6) were created from clusters of WT sequences based on percent similarity. Secondary consensus sequences (I &II) were created from the primary sequences; this multi-layered consensus building way continued until a final COBRA sequence was obtained. Each COBRA HA or NA nucleotide sequence was cloned into a pcDNA3.1+ plasmid vector, being truncated by removing the transmembrane domain and replacing with a T4 fold-on domain, an AviTag, and a 6× His tag for purification by immobilized metal affinity chromatography. The plasmid was transfected into human embryonic kidney 293T (HEK 293T) suspension cells for protein expression.

Fig. S2

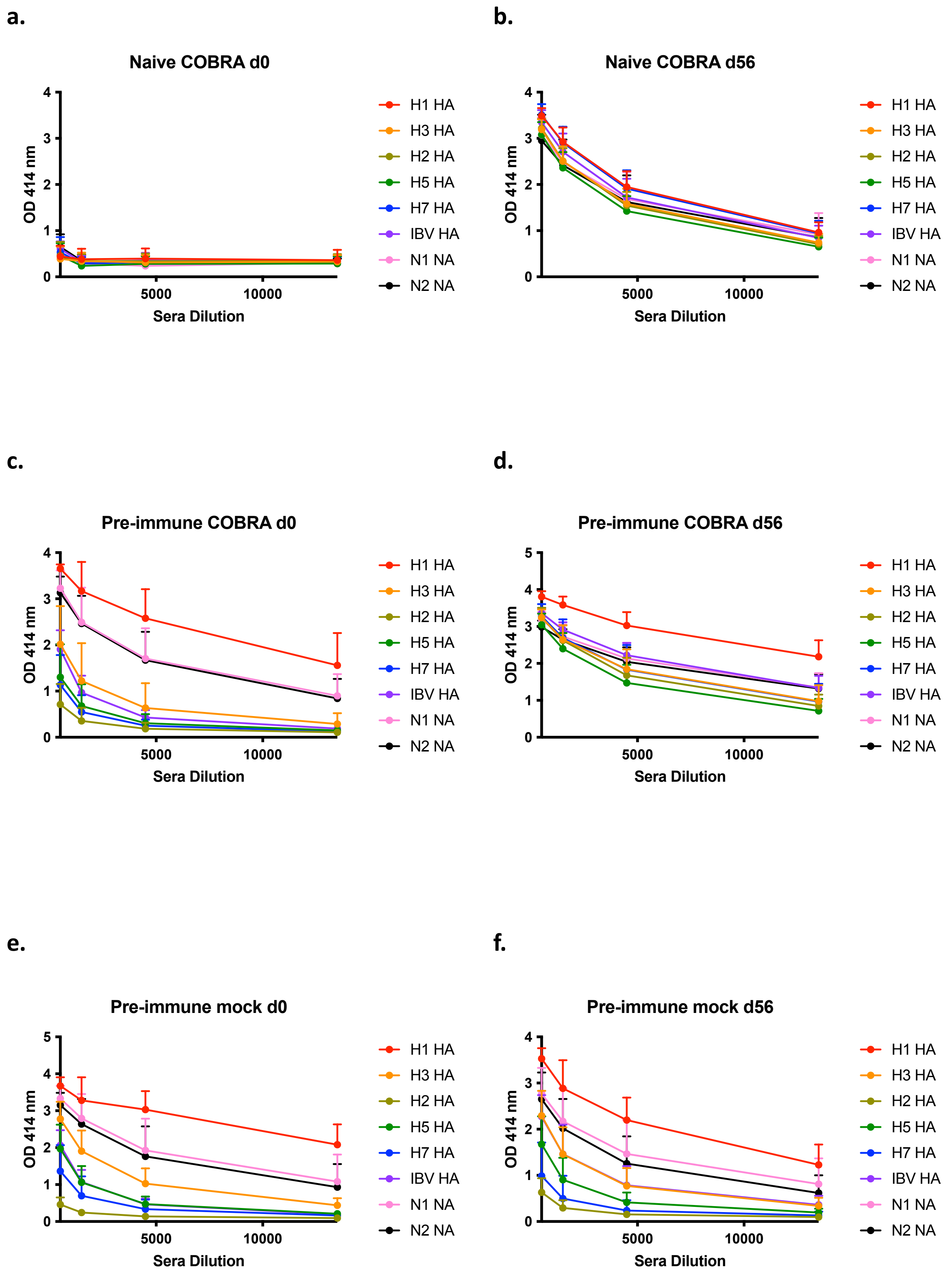

Supplemental figure 2: Total IgG antibody response is increased after octavalent COBRA vaccination in ferrets. Ferrets were vaccinated as described in Figure 1. Total IgG antibody titers were determined against each of the 8 COBRA components, as indicated on the x-axis, from sera collected before vaccination (d0, left) and after final vaccination (d56, right) from naïve ferrets given COBRA vaccination (a,b), pre-immune ferrets given COBRA vaccination (c,d), and pre-immune ferrets given mock vaccination (e,f).
